# The Roles of Autophagy and the Inflammasome during Environmental Stress-Triggered Skin Inflammation

**DOI:** 10.3390/ijms17122063

**Published:** 2016-12-09

**Authors:** Rong-Jane Chen, Yu-Hsuan Lee, Ya-Ling Yeh, Ying-Jan Wang, Bour-Jr Wang

**Affiliations:** 1 Department of Environmental and Occupational Health, College of Medicine, National Cheng Kung University, Tainan 70428, Taiwan; janekhc@gmail.com (R.-J.C.); bmm175@gmail.com (Y.-H.L.); linn7627@hotmail.com (Y.-L.Y.); 2 Department of Biomedical Informatics, Asia University, Taichung 41354, Taiwan; 3 Department of Medical Research, China Medical University Hospital, China Medical University, Taichung 40402, Taiwan; 4 Graduate Institute of Clinical Medicine, Taipei Medical University, Taipei 110, Taiwan; 5 Department of Occupational and Environmental Medicine, National Cheng Kung University Hospital, Tainan 70428, Taiwan; 6 Department of Cosmetic Science and Institute of Cosmetic Science, Chia Nan University of Pharmacy and Science, Tainan 71710, Taiwan

**Keywords:** inflammasome, autophagy, UVR, hexavalent chromium, TiO_2_/ZnO/Ag nanoparticles

## Abstract

Inflammatory skin diseases are the most common problem in dermatology. The induction of skin inflammation by environmental stressors such as ultraviolet radiation (UVR), hexavalent chromium (Cr(VI)) and TiO_2_/ZnO/Ag nanoparticles (NPs) has been demonstrated previously. Recent studies have indicated that the inflammasome is often wrongly activated by these environmental irritants, thus inducing massive inflammation and resulting in the development of inflammatory diseases. The regulation of the inflammasome with respect to skin inflammation is complex and is still not completely understood. Autophagy, an intracellular degradation system that is associated with the maintenance of cellular homeostasis, plays a key role in inflammasome inactivation. As a housekeeping pathway, cells utilize autophagy to maintain the homeostasis of the organ structure and function when exposed to environmental stressors. However, only a few studies have examined the effect of autophagy and/or the inflammasome on skin pathogenesis. Here we review recent findings regarding the involvement of autophagy and inflammasome activation during skin inflammation. We posit that autophagy induction is a novel mechanism inter-modulating environmental stressor-induced skin inflammation. We also attempt to highlight the role of the inflammasome and the possible underlying mechanisms and pathways reflecting the pathogenesis of skin inflammation induced by UVR, Cr(VI) and TiO_2_/ZnO/Ag NPs. A more profound understanding about the crosstalk between autophagy and the inflammasome will contribute to the development of prevention and intervention strategies against human skin disease.

## 1. Introduction

The skin is the largest organ of the body and serves as the first line of defense against many different environmental insults. Inflammatory skin diseases are the most common problem in dermatology. Acute inflammation can result from exposure to ultraviolet (UV) light, allergens, or toxic chemical irritants, whereas chronic inflammation results from a sustained immune cell-mediated inflammatory response. Tissue destruction can occur in chronic inflammation but is seldom found in acute inflammation. Nonetheless, the precise mechanisms regarding skin inflammation still need to be further elucidated [1]. Recently, a variety of studies indicated that autophagy plays essential roles in cell-fate decision, contributing to pathogen clearance, antigen presentation and inflammation. Those processes are important in the cellular homeostasis of skin [2]. Autophagy is a cellular self-consumption process for recycling of intracellular damaged proteins and organelles. Steady-state autophagy helps maintain homeostasis, while additional autophagy induced by environmental stress could serve as a cell-protective mechanism [2]. In addition, autophagy could also be induced by organelle stress and pathogen infection, and is considered to be closely associated with immune response and host defense [3].

The inflammasome forms a crucial part of the innate immune system. Canonical activation of the inflammasome is critical in promoting caspase-1-dependent maturation of the pro-inflammatory cytokines IL-1β and IL-18, as well as in inducing programmed cell death in response to pathogens and endogenous danger signals [4]. Inflammation forms an important part of the host defense system; however, untimely or persistent inflammation can also lead to inflammatory diseases. Thus, good control of the innate immune system is essential for the proper inflammatory responses. The molecular crosstalk between inflammation and autophagy is the focus of an emerging field of research that is essential for the understanding of multicellular organism homeostasis and how these processes influence a variety of pathological conditions. It has been reported that the inflammasome and autophagy often modulate each other via common inhibitory mechanisms that are controlled by different input pathways [5]. Thus, inflammasome components coordinate autophagy and vice versa, making the balance between both processes a fundamental player in cellular homeostasis [6]. As the accumulation of damaged intracellular organelles such as the mitochondria can cause inflammation, it is not surprising that autophagy serves as the anti-inflammatory machinery by eliminating dysfunctional organelles and participating in the immune response [7].

The induction of skin inflammation by environmental stressors such as ultraviolet radiation (UVR), hexavalent chromium (Cr(VI)), and TiO_2_/ZnO/Ag nanoparticles (NPs) has been demonstrated previously [8,9]. As a housekeeping pathway, cells utilize autophagy to maintain the homeostasis of an organ’s structure and function when exposed to environmental stressors [10]. However, only a few studies have examined the effect of autophagy and/or the inflammasome on the pathogenesis of skin diseases. In a recent review article, the authors highlighted the significance of autophagy regulation in skin diseases such as psoriasis, systemic lupus erythematosus, vitiligo, and infection [2]. They discussed the roles of autophagy in apoptosis, differentiation, inflammation and the immune response, thus highlighting the possible involvement of autophagy in skin disorders [2]. In our current review, we collect and integrate recent advances regarding autophagy and the inflammatory innate immune response, particularly the activation and regulation of the inflammasome. We posit that autophagy induction is a novel mechanism inter-modulating environmental stressor-induced skin inflammation. We also attempt to highlight the role of the inflammasome and the possible underlying mechanisms and pathways reflecting the pathogenesis of skin diseases induced by ultraviolet radiation (UVR), Cr(VI), and TiO_2_/ZnO/Ag NPs based on recent findings. A more profound understanding about the crosstalk between autophagy and the inflammasome will contribute to the development of prevention and intervention strategies against human skin disease.

## 2. Autophagy Intersects the Inflammasome

The first line of host defense against infection is equipped with pattern-recognition receptors (PRRs) that recognize pathogen-associated molecular patterns (PAMPs) and damage-associated molecular patterns (DAMPs) [11]. PRRs are currently classified into five families including toll-like receptors (TLRs), nucleotide-binding and oligomerization domain (NOD)-like receptors (NLRs), retinoic acid inducible gene-I (RIG-I)-like receptors (RLRs), C-type lectins (CTLs), and absent-in-melanoma (AIM)-like receptors (ALRs). TLRs and CTLs are located in the plasma membrane, while the NLRs, RLRs, and ALRs are intracellular PRRs. The recognition by NLRs of PAMPs and DAMPs from microbial structures, environment-derived molecules such as alum, asbestos, silica, alloy particles, UVR and skin irritants, and host cells including ATPs, cholesterol crystals, uric acid etc. leads to the activation of inflammatory responses [11]. Moreover, cellular signaling such as potassium (K^+^) efflux, pore formation in cell membranes, lysosomal membrane permeabilization (LMP), and release of lysosomal hydrolases and mitochondrial reactive oxygen species (ROS) are also responsible for inflammasome activation [12].

NLRs exhibit various functions when involved in inflammasome formation, signaling transduction, transcription activation, and autophagy. In response to PAMPs and DAMPs, NLRs recruit an ASC (apoptosis-associated speck-like protein containing a C-terminal caspase-recruitment domain, CARD) via a pyrin-pyrin domain interaction. Subsequently, pro-caspase-1 binds to ASC through CARD–CARD domains, which completes the formation of the inflammasome [11]. The inflammasome is an innate immune structure that is mainly activated in cells such as macrophages and it induces inflammation to protect the host from microbial infection [3]. The inflammasome complexes induce the cleavage of pro-caspase-1 to the active caspase-1 enzyme, which in turn cleaves the pro-inflammatory cytokines into their active forms such as IL-1β and IL-18, which are then secreted from the cells [5].

Among the skin epidermis, the keratinocyte is a fundamental cell type of the total cells and provides protective functions of the body against the entry of pathogens by forming an impenetrable boundary [13]. Recent reports showed that human keratinocytes express various PRRs including TLRs 1–10, except for 7 and 8 [13]. TLRs are also expressed in both innate and adaptive immune system-related cells, including macrophages, monocytes, dendritic cells and so on. It has been reported that TLRs play a role in UV-induced inflammatory processes both in rodent and human studies through unknown mechanisms [13]. In addition to TLRs, all the essential inflammasome components are expressed in cultured human primary keratinocytes including the NLRs (composed of 22 genes), the ASC, pro-caspase-1 and pro-IL-1 [14]. The expression of ASC, pro-caspase-1 and pro-IL-1β in human epidermis has also been confirmed [14]. Therefore, an alteration in inflammasome components has been reported as being associated with various diseases. For instance, the NLR family, pyrin domain containing 1 (NLRP1) and NLR family, pyrin domain containing 3 (NLRP3) receptors are two forms of the important inflammasome complexes in the UV-induced inflammatory processes of keratinocytes and they contribute to IL-1, IL-18, and possibly IL-33 activation [13]. *NLRP1* polymorphisms are significantly associated with various diseases such as systemic sclerosis, rheumatoid arthritis, and corneal intraepithelial dyskeratosis [11]. Single nucleotide polymorphisms (SNPs) in the *NLRP3* gene, *NLRP1*, and caspase recruitment domain-containing protein 8 (CARD8) which is the negative regulator of caspase-1 activity, are also associated with susceptibility with psoriasis [11]. In addition, *NLRP12* mutations are associated with atopic dermatitis (AD) [15]. *NOD2* gene mutations are also associated with Blau syndrome, atopic eczema, and AD [16,17]. An autoimmune disease, systemic sclerosis, which is expressed through skin fibrosis, systemic vascular alterations and collagen accumulation, has been reported to show stronger staining of NLRP3 and IL-1β cytoplasmic expression in the keratinizing squamous epithelium of skin [18]. Moreover, overexpression of important skin host defense TLRs is associated with some skin inflammatory diseases including AD, psoriasis, and acne vulgaris [19]. Among the inflammatory cytokines, the IL-1 family can induce a secondary cascade of mediators from keratinocytes and other cells, resulting in infiltration by inflammatory leukocytes, the induction of immunosuppression, DNA repair or apoptosis. Thus, the ability of keratinocytes to produce pro-inflammatory cytokines can influence the resident dermal leukocytes, including dermal dendritic cells, mast cells, macrophages and endothelial cells, thus impacting the immune response both locally as well as systemically [13].

The inflammasome regulates host defense and repair, while over-activation is linked to many inflammatory diseases. In this context, the negative regulation of the inflammasome has important controlled mechanisms to prevent potential damage and preserve homeostasis [5]. A comprehensive review regarding negative regulation of the inflammasome has been reported and the author indicated that negative regulation mechanisms of inflammasome activity including autophagy, endogenous proteins, phosphorylation of inflammasome components, inhibition by immune mediators, negative regulation by heat shock chaperons and ubiquitination, regulated by microRNAs (miRNAs, a group of RNA nucleotides that modulate mRNA translation and degradation), and by exogenous pathogens [20]. In addition, lysosome rupture and release of lysosomal cathepsins have been shown to induce NLRP3 activation; pharmacologic inhibition of cathepsin activity could attenuate LMP-induced inflammasome activation [21]. Interestingly, a recent study indicated that high-level lysosome disruption can suppress NLRP3 inflammasome activation [22]. Katsnelson et al. provided new insights that increasing LMP resulted in NLRP3 ubiquitination, and LMP-induced Ca^2+^ influx attenuated the association of NLRP3 with ASC [22]. These studies indicate that lysosomal disruption can either activate or suppress inflammasome activation depending on different levels of LMP and different cellular contexts [22].

Among above mentioned negative regulation mechanisms, autophagy is currently the most fully characterized negative regulation mechanism in the regulation of the inflammasome, pathogen clearance, and antigen presentation, and is also essential for cellular homeostasis in the skin [2]. Autophagy is a highly regulated mechanism that occurs in four phases: induction, nucleation, elongation, and fusion [5]. In the induction step, autophagy is initiated by many possible signaling cascades involving class III phosphatidylinositol-3-kinase, 5′-adenosine monophosphate activated protein kinase (AMPK), elongation initiation factor 2α (eIF2α), p53, c-Jun N-terminal kinase 1 (JNK1), inositol-phosphate requiring enzyme 1 (IRE-1), inositol-triphosphate receptor (IP3R), or intracellular calcium [11]. The second step is vesicle nucleation, which results in the assembly of an autophagophore to engulf the cytosolic cargo [5]. The third step is autophagophore elongation via a ubiquitin E3 ligase complex consisting of autophagy proteins (Atgs) including Atg5, Atg12, and Atg16L1, which conjugates phosphatidylethanolamine (PE) to light chain 3 (LC3) [11]. During the final fusion step, the autophagosome fuses with the lysosome to create an autolysosome and allow lysosomal enzymes to degrade the sequestered cytoplasmic materials for reuse [5].

**Scheme 1:**
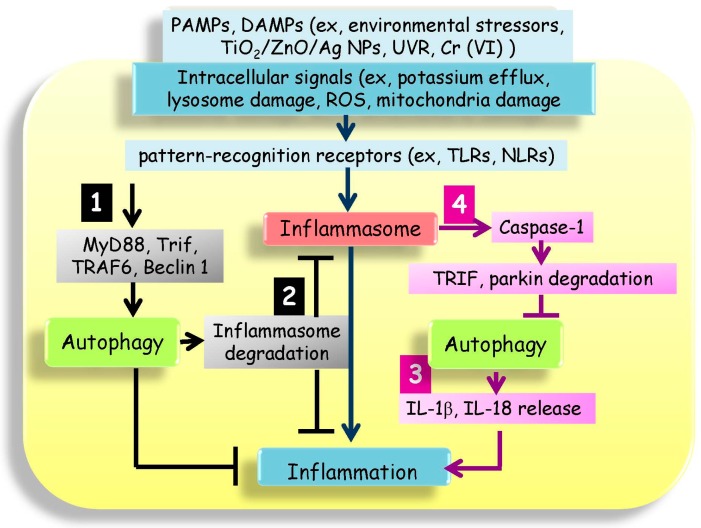
The mutual regulation mechanisms of autophagy and the inflammasome. In response to PAMPs, DAMPs (such as TiO_2_/ZnO/Ag NPs, UVR and Cr(VI)), and cellular signaling such as potassium efflux, lysosome damage, and release of mitochondrial ROS, PRRs including TLRs and NLRs are activated and then recruit ASC and pro-caspase-1 protein to form the inflammasome. The inflammasome is critical in promoting caspase-1 dependent maturation of the inflammatory cytokines to induce tissue inflammation. Autophagy is one of the regulation mechanisms of the inflammasome and mutual regulation mechanisms exist between autophagy and the inflammasome, for example: (1) Autophagy could be activated by inflammasome sensors against inflammation; (2) Autophagy negatively regulates inflammasome components and inflammatory cytokines through autophagy-dependent degradation mechanisms; (3) Autophagy could increase the release of pro-inflammatory cytokines such as IL-1β and IL-18; and (4) The inflammasome negatively regulates autophagy through caspase-1 release and degradation of Trif or parkin. ASC: apoptosis-associated speck-like protein containing a C-terminal caspase-recruitment domain; Cr(VI): hexavalent chromium; DAMPs: damage-associated molecular patterns; NLR: nucleotide-binding and oligomerization domain (NOD)-like receptors; NPs: nanoparticles; PAMPs: pathogen-associated molecular patterns; PRRs: pattern recognition receptors; TLR: toll-like receptors; Trif: toll/interleukin receptor domain-containing adaptor protein-inducing interferon β; ROS: reactive oxygen species.

Defects in autophagy are related to several diseases such as cancer, neurodegenerative diseases, autoimmune skin disorders, infectious skin diseases, and skin cancer diseases [2]. For instance, in chronic autoimmune skin diseases, psoriasis has been reported that could be associated with SNPs in the *ATG16L1* gene [23], and systemic lupus erythematosus (SLE) could be associated with a genetic variant within or near *ATG5* gene [24]. In infectious skin diseases, autophagy plays an important role in degradation of microbes. Genetic disruption in *ATG5* could reportedly increase herpes simplex virus (HSV) infections of the skin and mucous membrane [25]. The activity of autophagy and the inflammasome are both important cellular responses to stresses. Prior evidence indicates that these two responses mediate dynamic crosstalk to maintain tissue homeostasis. However, the mutual regulation mechanisms of autophagy and inflammasome are not fully understood. Based on the current findings, the possible mutual regulation mechanisms are shown in Scheme 1.

### 2.1. Autophagy Could Be Activated by Specific Inflammasome Sensors

Recent studies suggest that the primary cellular sensors for PAMPs, TLRs, can regulate autophagy through the activation of downstream signaling processes in macrophages and other cell types [5]. Sci et al. indicated that TLR signaling induces interaction of myeloid differentiation primary response gene-88 (MyD88) and toll/interleukin receptor domain-containing adaptor protein-inducing interferon β (Trif) with Beclin 1 leading to autophagy induction [26]. The author further indicated that TLR4 could trigger tumor necrosis factor (TNF) receptor-associated factor 6 (TRAF6) activation followed by ubiqutination of Beclin 1 and autophagosome formation [27]. Other inflammasome components were also reported to be able to bind to and regulate autophagy proteins to initiate autophagophore formation. For instance, Byrne et al. indicated that macrophages enlist autophagy by NLR proteins neuronal apoptosis inhibitory protein 5 (NAIP5) and NLRC4 and pro-caspase-1 to act against infection-induced pyroptosis [28].

### 2.2. Autophagy Regulates Inflammasome Inactivation and Degradation

Autophagy also negatively regulates the inflammasome through autophagy-dependent degradation of inflammasome proteins and pro-inflammatory cytokines [5]. Autophagosomes can degrade inflammasome inducers such as depolarized mitochondria, therefore preventing ROS production. Since ROS production is important during inflammasome stimulation, when autophagy is defective, damaged mitochondria release mtROS that can stimulate NLRP3 inflammasome activation [29]. In addition, autophagosomes can sequester and degrade inflammasome components, including NLRP3, ASC, and caspase-1. For instance, ASC-containing inflammasome are redirected towards autophagolysosomes in THP-1 and primary human macrophage cells dependent on both Beclin-1 and p62, a protein that acts as an ubiquitin sensor and that specifically recruits ubiquitinated proteins for degradation [30]. Therefore, ubiquitinated ASC is degraded by selective autophagy, resulting in inflammasome suppression and inducing IL-1β degradation [30]. NLRP3 is also ubiquitinated and experiences a loss of activity through recruitment to autophagosomes [31]. However, the mechanism of ASC and NLRP inhibition via ubiquitination and autophagy remains unknown, thus further investigation is imperative to elucidate the molecular mechanisms of how autophagy may work as a potential “off switch” for the activated inflammasome.

In addition to upstream activators of the inflammasome, previous reports indicate that IL-1β is sequestered in the TLR-stimulated autophagosomes and the protein levels of pro-IL-1β decrease when autophagy is induced by rapamycin [32]. This suggests that TLR stimulation induces pro-IL-1β expression; meanwhile, autophagy is also activated, thereby limiting the amount of available pro-IL-1β protein [21]. IL-1β secretion was also apparently increased by the inhibition of autophagy by 3-methyladenine (3-MA) treatment, the expression of the Atg4B dominant negative mutant, the deletion of LC3B, the deletion of Atg16L1, or the depletion of Beclin1 [5].

### 2.3. Autophagy Regulates Biogenesis and Release of Inflammatory Cytokines

Although autophagy is reported as a potent suppressor of inflammation, autophagy is also involved in the unconventional secretion of pro-inflammatory cytokines. In a recent report, the authors suggested that mature IL-1β may trigger the autophagy machinery for secretion in a non-canonical secretory pathway [33]. Using Alternaria extract, induced IL-18 and inflammation in airway epithelial cells has been shown to be autophagy dependent but caspase-1 independent [34]. The pro-inflammatory roles of autophagy have also been reported in normal hepatocytes induced by hepatitis B virus (HBV); small interfering RNAs targeting ATG5 and ATG7 abolished autophagy-induced activation of NF-κB and the production of IL-6, IL-8, and CXCL2 [35]. All these studies demonstrated that autophagy could increase the release of pro-inflammatory cytokines in specific biological contexts; however, the precise mechanism still requires further investigation.

### 2.4. Inflammasome Possesses Inhibitory Effects on Autophagy

It has been reported that mutual antagonism mechanisms exist between autophagy and inflammasome, showing that inflammasome displays an inhibitory effect on autophagy. Jabir et al. indicated that *Pseudomonas aeruginosa* induced macrophage NLRC4 inflammasome activation; NLRC4 activated caspase-1 then directly cleaved Trif to reduce Trif-mediated autophagy [36]. Similarly, AIM2 and NLRP3 also negatively regulated autophagy through caspase-1 release and parkin (a key mitophagy protein) inactivation, leading to amplify mitochondria damage and pyrotosis [37]. Furthermore, NLRP4 has also been reported to negatively regulate autophagy through interaction with Beclin 1 [38].

## 3. Cr(VI)-Induced Skin Inflammation

Cr is widespread in the environment, and highly toxic Cr(VI) is used in many industrial processes. Cr-induced allergic contact dermatitis is an important factor in occupational skin disease. Recent studies have also indicated that some metal-implant debris (e.g., soluble CoCl_2_, CrCl_3_, MoCl_5_, and NiCl_2_ ions and cobalt alloy particles) can activate the inflammasome pathway [39]. We have previously demonstrated that Cr(VI) can increase ROS formation, activate the Akt, NF-κB, and MAPK pathways, and increase the production of cytokines (TNF-α, IL-1α) both in HaCaT cells and in a guinea pig (GP) model. The release of these cytokines from keratinocytes is considered key in the pathogenesis of contact hypersensitivity [40]. In addition, the induction of apoptosis, autophagy and ROS was observed after treatment with Cr(VI). HaCaT cells pre-treated with *N*-acetyl cysteine exhibited a decrease in apoptosis and autophagy, which could affect cell viability, indicating that ROS-induced cell death and cytokine expression contributed to Cr hypersensitivity [41]. Caicedo also confirmed that Cr and cobalt-Cr-molybdenum (Co-Cr-Mo) alloy particles induce both upstream and downstream components of inflammasome activation, including NADPH/ROS-, active caspase-1-, Nalp3-, and ASC-dependent IL-1β secretion in human macrophages, leading to mature IL-1β secretion and a wide pro-inflammatory response [42]. In addition, Xie et al. have confirmed that Cr(VI) induced a decrease in mitochondrial membrane potential, leading to apoptosis in hepatocytes, and that autophagy can remove damaged organelles, thus protecting cells from mitochondria-dependent cell death [43]. In this process, ROS production triggered Cr(VI)-induced mitochondria-dependent apoptosis. These studies supported Cr(VI) as inducing inflammasome activation and autophagy in the skin. It could be suggested that autophagy acts as a protective mechanism against Cr(VI)-induced inflammation, however, the complex relationship between skin inflammation induced by Cr(VI) and the role of autophagy and inflammation needs further study.

## 4. The Possible Role of the Inflammasome and Autophagy in UVR-Induced Skin Inflammation

UVR is a common environmental stressor that leads to a series of morphological, ultrastructural and physiological alterations, and carcinogenesis in human skin [13]. The UVR that reaches the earth is comprised of UVA (320–400 nm), while UVB (290–320 nm) makes up the remainder (3%–6%). UVR-induced skin damage occurs through direct and indirect mechanisms that cause the physical and metabolic disruption of epidermal cells. Triggering an innate immune response and releasing immune-regulatory cytokines and chemokines, including IL-1β, IL-6, TNF-α, and IL-8, plays an important role in the pathogenic process of UVR [44]. One of the earliest responses to UVR-induced damage is made by viable keratinocytes forming the inflammasome, resulting in the accumulation and release of highly conserved endogenous cellular constituents termed “alarmins” [13]. Alarmins are extracellular DAMPs including IL-1α and IL-33 that induce inflammation and could be perceived by cells as potent “danger” signals that sound the alarm to avoid tissue damage [13].

How UVB irradiation triggers inflammasome activation remains unclear [14]. It has been reported that NLR-family proteins represent a first line of defense against tissue injury induced by UVR [14]. Consistently, Feldmeyer and colleagues have shown that UV-irradiated keratinocytes secreted NALP1, ASC, IL-1β, and caspase-1 [45]. The studies demonstrated a prominent role for NLR-family proteins in sunburn-associated inflammation [14]. Salzer et al. also confirmed that exposure to UVB results in inflammasome-mediated processing and IL-1β secretion; meanwhile, numbers of human cathelicidin LL-37-regulated P2X7 receptors and UV-induced IL-1β secretion were shown to increase in keratinocytes [44]. UVB and LL-37-induced P2X7 receptor activation increased intracellular calcium concentrations, which triggered inflammasome activation and IL-1β release [44]. Using Ginsenoside Rg3 (the major bioactive ingredient of Panax ginseng) as an antioxidant to regulate inflammasome can block nitric oxide (NO) production and thus decrease the NLRP3 inflammasome in macrophage and HaCaT cells following UVB exposure [46]. It has been shown that ROS are involved in UVB-induced activation of the NLRP3 inflammasome [46]. Clearly, much remains to be learned about the mechanisms of inflammasome activation and its pathophysiological consequences in the context of UVR-induced skin damage.

Keratinocytes respond to UV-induced damage either by repairing or tolerating it, ultimately undergoing cell death [9]. Normal keratinocytes have high autophagic activity, suggesting that autophagy in keratinocytes is involved in host defense against exogenous pathogens and contributes to the innate immune defense by eliminating intracellular viruses and microbes [9]. The features of autophagy have also been observed in UV-treated cells, suggesting that autophagy might be one of the cytoprotective mechanisms against UV-induced damage in keratinocytes [47]. Mechanistic studies have indicated that the UV radiation resistance-associated gene (UVRAG), a tumor suppressor involved in autophagy, is recruited to UV-damaged foci and thus activates the photo lesion repair mechanism [48]. Recently, the Rac1-Armus-Rab7 axis was shown to participate in regulating the recruitment of LC3 to autophagosomes in keratinocytes [49]. The inactivated Armus delays autophagy flux by blocking the initiation of phagophores, and activated Rac1 competes with LC3 for the Armus to prevent its recruitment to autophagosomes [9]. Furthermore, a newly discovered autophagy-related gene, autophagy and beclin 1 regulator 1 (AMBRA1), is a crucial regulator of autophagy that plays an important role in the regulation of UV-induced premature senescence and autophagy [50]. In addition, the ATG7-deleted keratinocytes irradiated by UVA showed defective clearance of p62 and the elevation of nuclear factor-like 2 (Nrf2) target gene expression [51]. Besides the canonical autophagy pathway, ATG5/ATG7-independent but Beclin1-dependent autophagy has been described in keratinocytes [52]. These studies suggested that the canonical and non-canonical autophagy pathways in keratinocytes participate in UVR-induced autophagy.

## 5. Possible Role of Inflammasome and Autophagy in Nanoparticle-Induced Skin Damage

The rapid development of nanotechnology has led to the widespread application of nanomaterials in various industrial sectors (medicine, manufacturing, electronics etc.). Due to their small size, NPs have the ability to enter through the airways and skin, threatening human health. The use of TiO_2_ and ZnO NPs in skincare products is extremely common. For example, about 70% of all sunscreens containing TiO_2_ and 30% of those containing ZnO are formulated with NPs [8]. Based on existing scientific evidence, ZnO and TiO_2_ NPs have been considered as safe materials when applied in dermatologic emollients, sunscreen and other topical products. A recent review article indicated that TiO_2_ and ZnO NPs cannot penetrate the skin and exert adverse effects in most circumstances [8]. Among rats, pigs and human skin analysis, TiO_2_ NPs have only been observed in the epidermis and hair follicle [53,54,55,56]. No transdermal absorption was detected when porcine skin was exposed to larger size (90–460 nm) of TiO_2_ NPs in water/oil. However, NPs could penetrate deeper into UVB-damaged skin [57]. Similar findings were found in the study of ZnO NPs in skin penetration. ZnO NPs (despite having a particle size of less than 20 nm) could not penetrate healthy and intact human skin [54,58]; whereas ZnO NPs could penetrate into skin in the UVR-caused skin damage model [59]. However, the quantity of NPs that penetrate the skin and the health effects are still quite controversial. Different circumstances and conditions may result in different reactions. Therefore, Larese Filon et al. indicated that those NPs are generally very stable and can be considered as having a low risk of skin penetration or permeation, with such permeation only being possible with NPs < 10 nm and when the skin is damaged [8]. Ag NPs are widely used in many consumer products. Skin exposure to Ag NPs is mainly due to their increasing inclusion in textiles, burn creams, wound dressings, and catheters. The interaction of NPs with the skin is still a matter of investigation by researchers, since the health hazards involved following the transdermal flux of NPs has opened the debate on toxicological, therapeutic, and drug-delivery issues that have still to be resolved [60].

In this section, we have collected and organized recent studies about the mechanisms of NP-induced skin damage and inflammation and focus on TiO_2_, ZnO and Ag NPs that can potentially come into contact with human skin during their manufacture and use in commercial products.

### 5.1. Titanium Dioxide Nanoparticles (TiO_2_ NPs)

TiO_2_ is widely applied in the form of microparticles and NPs in consumer goods, including cosmetics, pharmaceuticals and foods, to produce a white color and prevent UVR damage [61]. The biological effects of TiO_2_ NP exposure are still unclear, yet many TiO_2_ NP-induced toxic effects have been reported. TiO_2_ NPs can cause several adverse effects on mammalian cells, including ROS production [62,63], DNA damage responses [64], proliferation, and the induction of apoptosis [65,66]. Hiroike et al. suggested that acicular, but not globular TiO_2_ NPs stimulate keratinocytes to produce pro-inflammatory cytokines such as IL-1α, IL-1β, IL-6, TNF-α, and IL-8 [67]. The increased production of these cytokines leads to cutaneous inflammatory responses, as typically seen in contact dermatitis [68]. Recently, TiO_2_ NPs were reported to stimulate inflammasome in murine dendritic cells [69] and may activate inflammasome by acting as crystals in keratinocytes [70].

TiO_2_ NPs also have an impact on certain types of energy metabolism that are known to be associated with the cellular stress response and mitochondrial function. The treatment of human keratinocytes with TiO_2_ NPs resulted in cellular stress and reduced metabolic capacity. TiO_2_ NPs did not affect cell cycle phase distribution, nor was there cell death in the experimental condition, but the uptake of NPs into the keratinocytes was restricted to autophagosomes throughout the cytoplasm [71]. The same experimental results were described in recent reports which Zhao et al. observed the induction of autophagy during TiO_2_ NP exposure and speculated that autophagy allowed keratinocytes to survive in response to the stress induced through the interaction with TiO_2_ NPs [72]. Lopes et al. also demonstrated that exposure to TiO_2_ NPs led to a dose-dependent increase in the autophagic effect under non-cytotoxic conditions, but the high-dose treatment appeared to impair the degradation of autophagic substrates more than the low-dose did over time [73]. This disruption of autophagy flux might initiate many adverse biological effects. Therefore, through interfering with autophagosome fusion with lysosomes, TiO_2_ NP exposure resulted in the accumulation of inflammasome and in sustained IL-1β production, leading to continual inflammatory responses [74]. To date, only very few studies have shown that TiO_2_ NPs can penetrate the skin and translocate to other tissues. Both coated and non-coated TiO_2_ NPs have been shown not to penetrate through the epidermis and into the viable skin layers in normal skin [75]. However, another study revealed that TiO_2_ NPs did penetrate into deeper areas of the stratum corneum in psoriatic skin than in healthy skin [76]. Hence, we still need to consider all possible conditions of exposure to TiO_2_ NPs and their possible effects on skin inflammation.

### 5.2. Zinc Oxide Nanoparticles (ZnO NPs)

In a manner similar to that of TiO_2_, ZnO is well known and frequently used metal oxide in many diverse products such as paints, coatings, and in cosmetic products such as sunscreen formulations due to its antimicrobial or UV-protection properties [77,78]. The safety of ZnO NPs when used as a UV protector has been of continuing controversy, with many of the safety concerns mainly based on cell culture and animal data as well as human studies [79]. Most of the studies have found that the ZnO NPs remain on the skin surface and do not enter the viable epidermis [8,57,80]. However, in most cases, a damaged skin barrier allows for the penetration and permeation of NPs or can increase their absorption. For instance, only ZnO NPs can reach into the deep layers of allergic skin whereas bulk of the ZnO stays in the upper layers of both damaged and allergic skin. In the atopic dermatitis mouse model, topically applied ZnO NPs suppress allergen-induced skin inflammation but induce vigorous Immunoglobulin E (IgE) production. Jeong et al. utilized HaCaT cells and primary keratinocytes to investigate the inflammatory response induced by ZnO NPs. They indicated that ZnO NPs might induce an inflammatory response via the ROS/extracellular signal-regulated kinase (ERK)/early growth response-1 (Egr-1) pathway in human keratinocytes [81]. In addition, Heng et al. reported that ZnO NPs can induce inflammation in the human bronchial epithelial cell line [82]. Interestingly, although ZnO NPs have been reported to induce inflammation in different cell types, the induction of the NLRP3 inflammasome is possibly not required for the inflammatory response in primary human keratinocytes and myeloid cells [83].

Recent studies have also indicated that ZnO NPs promote autophagy in cells. Johnson et al. determined that ZnO NPs release free Zn^2+^ and it is subsequently taken up by cells, resulting in cell death. Meanwhile, ZnO NPs triggered ROS release, in turn initiating autophagic death in immune cells [84]. In addition, treatment of normal mouse skin epidermal cells with ZnO NPs also confirmed that ZnO NPs lead to cell death through autophagic vacuole accumulation and mitochondrial damage in normal skin cells [85]. These findings may provide some possible answers regarding the adverse effects of ZnO NPs via inflammation and an autophagy defect, which could provide new insights into the ZnO NPs that are commonly used in cosmetic products.

### 5.3. Silver Nanoparticles (Ag NPs)

Skin exposure to Ag NPs occurs mainly as a result of their incorporation in textiles, burn creams, jewelry, wound dressings and personal hygiene products. The Ag ions released from textiles and wound dressings can interact with the skin and penetrate it. The overt manifestation of silver metal intoxication is the development of characteristic, irreversible bluish-black discoloration as a result of Ag deposition in the cutaneous tissues (argyria) and/or the eyes (argyrosis) [86]. Samberg and co-workers demonstrated the localization of all Ag NPs in the cytoplasmic vacuoles of human epidermal keratinocytes (HEKs). Consequently, Ag NPs significantly increased cytokine concentrations (IL-1β, IL-6, IL-8, and TNF-α) and induced inflammation in porcine skin [87]. In a more recent study, Ag NPs were shown to temporarily impair the biological barriers in the skin of the external ear canal, the mucosa of the middle ear, and inner ear via CD68 upregulation and TLR-4 activation [88]. Recent studies also indicated that the Ag NPs induced inflammasome formation that triggered the release of the key pro-inflammatory cytokine, IL-1β, in liver cancer cells [89,90]. Through downregulating the endoplasmic reticulum (ER) stress sensor activating transcription factor-6 (ATF-6), Simard et al. have demonstrated that ATF-6 is an important target of Ag NPs, leading to activation of the NLRP3 inflammasome and pyroptosis [91]. These studies implicated that the possible effects of Ag NPs in skin inflammation were through inflammasome activation.

Our recent work firstly identified the correlation between Ag NP exposure and autophagy activation and speculated that LC3 and p62/SQSTM1 protein accumulation resulting from defective autophagy may also potentially account for Ag NP cytotoxicity [92]. Although a few skin studies have referred to the relationship between Ag NPs and autophagy, Ag NPs are still quite versatile in skin applications and there is a need to clarify the relevant cellular mechanisms to elucidate the mechanisms of skin inflammation induced by Ag NPs.

## 6. Conclusions and Perspectives

The epidermis mainly consists of multi-layered continuously renewing keratinocytes that form the epidermal barrier which necessarily contributes to the defensive responses against various environmental stressors that we have mentioned above. Inflammasomeare multiprotein complexes that act as a major mediator for inflammatory responses as well as being involved in skin inflammation. In response to DAMPs, the inflammasome is activated and acts as an early protective mechanism to initiate the inflammatory responses required for wound-healing and maintaining the integrity of epidermal barrier [13]. The released cytokines (IL-18, IL-1β, and IL-33) act locally and systemically to activate specific cellular immune systems. While the mechanisms of inflammasome activation involved in UVR and environmental stressors-induced skin inflammation are important, much remains to be learned about this interesting mechanism for skin inflammation in the context of host defense and responses to tissue injury.

Current studies indicate that autophagy is important for the regulation of inflammation through disruption of multiple steps of inflammasome activation, therefore preventing inflammation [93]. Moreover, autophagy could downregulate pro-inflammatory cytokine production, exert a protective role in inflammatory diseases, and clear cellular materials such as damaged mitochondria to maintain cellular homeostasis; all these effects play a supporting role in the protection against inflammation associated skin diseases [3]. Therefore, autophagy seems to be a promising therapeutic target for treatment of inflammasome-related inflammatory skin disorders. For example, recent studies suggest that the vitamin D analogue calcipotriol could induce autophagy, and it has been used in the treatment of various skin diseases such as psoriasis, epidermolytic hyperkeratosis, and lamellar ichthyosis [2]. Autophagy induced by resveratrol could reduce UVB-induced apoptosis in keratinocytes, thus reducing malignant transformation [94]. Other natural components such as disaccharide trehalose and apigenin are also reported that can induce autophagy to against UVB-induced cell death [95,96]. In addition, a natural cyclopeptide, roseotoxin B, has also been demonstrated to trigger autophagy to overcome picryl chlororide-induced contact hypersensitivity in mice [97]. Recently, miRNAs have been reported to be involved in autophagy regulation [9]. Experiments demonstrated that miR-23a overexpression could reduce autophagy, and therefore regulate UVB-induced premature senescence [50]. Another miRNA, miR-34c-5p, was reported to enhance UVB-induced premature senescence in fibroblasts via regulation of some senescence-related molecules, whereas the involvement of autophagy is unclear [98]. Due to limited reports devoted to the regulation of miRNAs and autophagy, their precise mechanism has yet to be determined. This review provided the current intersection between the inflammasome and autophagy that could further highlight the possible regulation in response to environmental stressors in skin. Moreover, the notion of autophagy plays an essential role in inflammasome regulation, providing a molecular basis for innovative drug development in skin disorders.
